# Maxillofacial Reconstruction With Three Dimensional Resin Bone Substitutes as an Alternative to Transition Group of Metals: A Structured Review

**DOI:** 10.7759/cureus.57396

**Published:** 2024-04-01

**Authors:** Ashok V, Vaishnavi Rajaraman, Padma Ariga, Deepak Nallaswamy

**Affiliations:** 1 Department of Prosthodontics, Saveetha Dental College and Hospitals, Saveetha Institute of Medical and Technical Sciences, Saveetha University, Chennai, IND

**Keywords:** transition metals, polymethyl meth-acrylate, polylactic acid (plga), bone graft substitutes, bone growth

## Abstract

In recent years, novel technologies and techniques have allowed today the production of controlled architecture materials. Although autogenous bone graft substitutes remain the gold standard, enormous defects require supplementary alloplastic substitutes for reconstruction. Polymers have lately been explored for the same purpose and their biological performance has been under research since the last decade. The aim of this review is to analyse maxillofacial reconstruction with three-dimensional resin bone substitutes. A Problem Intervention Comparison Outcomes (PICO) analysis was done and a search was carried out in the Cochrane Database, PubMed, Google Scholar etc databases and a hand search was done to collect the related literature. All articles for maxillofacial reconstruction with three-dimensional resin bone substitutes were scrutinised. The manuscripts published from 1990 till May 2021, were included in this review. A total of 106 articles were obtained from a PICO-based keyword search, and 91 manuscripts were retrieved after excluding the duplicates. Out of these 57 manuscripts were excluded on the basis of title and abstract. From the remaining 34 studies, 17 were excluded after reading the full text based on the inclusion and exclusion criteria. During data extraction, four studies were removed and finally, 13 studies were included in this research. From this scoping review, we could conclude that polymethylmethacrylate and polylactic acid formulations are very promising resin bone substitutes for 3-dimensional reconstruction of maxillofacial defects. However, rigorous long-term clinical trials are needed to validate this conclusion.

## Introduction and background

Bone substitutes are popularly used in neurosurgery, orthopedics, and dental surgical procedures [1]. Their use has escalated with the latest development of injectable graft materials [2]. In craniofacial defects, transition metals are an age-old used biomaterial with long-term survival [3]. Recently, the most promising alloplastic materials commonly used include Poly Lactic acid Glycolic Acid (PLGA) and Poly Methyl Meth Acrylate (PMMA) [4]. PGLA-based scaffolds have suitable bone regenerative capacity and exhibit excellent prospects for bone repair [5,6]. Porous PMMA is a biocompatible material produced from PMMA powder and MMA liquid in which carboxymethylcellulose gel in the aqueous form is dispersed to generate pores that enable ingrowth resulting in improved acceptance of the graft [7-9]. 

In recent years, unique and innovative technologies have enabled the production of controlled architecture grafts. Various studies have been done time and again to experiment on the coating of implants [10,11], bone substitutes, etc. Studies show promising results for alloplastic substitutes as successful bone substitutes for enhanced regenerative properties. PLGA bio-polymers have been instrumental in applications in tissue engineering [12-15]. Many challenges faced in this research include potential risks like immunogenicity, toxicity, and prone to infections. Despite being biocompatible, the practical application of pure PLGA for bone regeneration in clinical scenarios is impeded by various drawbacks, including reduced osseoconductivity and unsatisfactory mechanical properties for load-bearing areas.

Today, numerous bone graft materials are commercially procurable. Nevertheless, there is no clarity in the consensus regarding the use of suitable bone graft material in specific practical configurations like maxillofacial prostheses [16,17]. Furthermore, the outlooks of alloplastic bone substitutes are also based on the trends among countries and their current market status. About 36 alloplastic graft materials, since 1980, have been approved by the Ministry of Health & Welfare database in Korea. Since 1996, the United States Food and Drug Administration has recorded 87 alloplastic materials approved for use as grafts in the United States. According to the Pharmaceuticals and Medical Devices Agency records, since 2004 about 10 materials have been permitted for use in Japan. The approved alloplastic bone substitutes include hydroxyapatite, biphasic calcium phosphate, and β-tricalcium phosphate. The development of new bone alloplastic grafts is ongoing in the biomaterial research sector. In the future, alloplastic bone grafts could be the first choice instead of autogenous bone, provided they pass the necessary safety and quality standardization. They may provide enhanced osteoconductive and osseoinductive effects in combination with better handling and optimal resorption rate. The objective of this review is to evaluate the existing evidence on maxillofacial reconstruction with three-dimensional (3D) resin bone substitutes.

## Review

Structured question

Is there a significant difference between polymethyl methacrylate and polylactic acid when used as a bone substitute in discontinuity defects? Problem Intervention Comparison Outcomes (PICO) analysis: P - discontinuity defect, I - polymethyl methacrylate, C - polylactic acid, O - bone gain.

Literature sources used

For the analysis of studies retrieved for this scoping review, the search methodology used suitable keywords in the PICO format. They describe population, intervention, comparison, and outcome. To obtain articles electronically, within each group, OR boolean was used and the searches of individual groups were coupled using AND boolean. The electronic databases included were: PubMed, Google Scholar, Cochrane Database of Systematic Reviews, Embase, Scopus, Web of Science, and Science Direct. 

Search terms

Population: Discontinuity defect, Lateral Discontinuity defect, medial discontinuity defect, Mandibular discontinuity defect, Mandibular discontinuity, Mandibular disconnection defect, Mandibular disconnection, Disconnection defect, Lateral Disconnection defect, Population, Patients, Population, Participants, defect, Male, female, Mandible, Break, Segmental resection, Mandibular Segmental resection.

Intervention: PMMA, poly methyl methacrylate, poly methyl methacrylate resin, PMMA resin.

Comparison: polylactic acid substitute, polylactic acid copolymer, polylactic acid substitutes, polylactic acid copolymers, poly(lactic-co-glycolic acid), polyethylene, porous polyethylene, polymer bone substitutes, polycaprolactone.

Outcome: bone gain, bone turnover, bone formation, bone regeneration, bone fill, bone augmented, newly formed bone, bone growth.

Article eligibility criteria

The inclusion criteria included articles containing discontinuity type of defect, articles with polymer-based bone substitutes, randomized control trials, cohort studies, prospective or retrospective studies, and articles including composite graft materials with the intervention and/or comparison. The exclusion criteria were any other defect apart from discontinuity defect, any other alloplastic bone substitutes, case series, reviews, case reports, and articles containing hip arthroplasty.

Results

**Figure 1:**
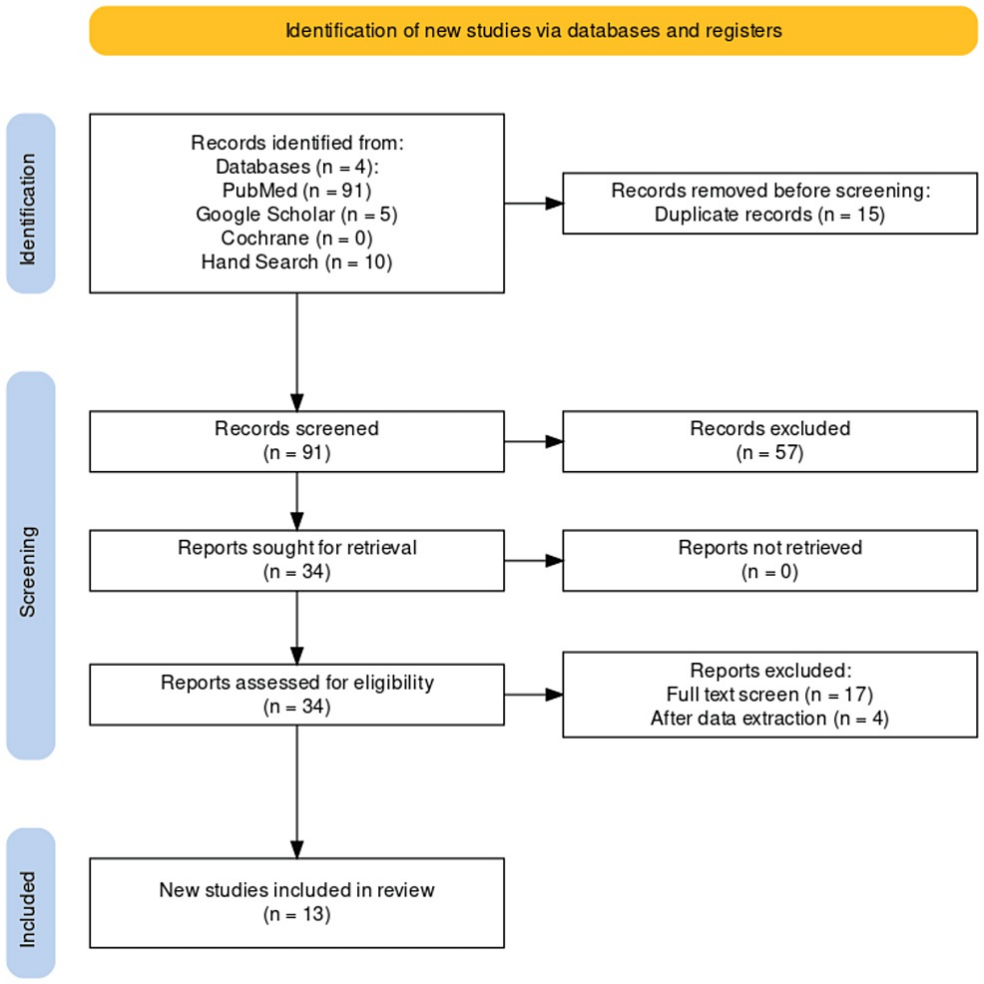
PRISMA flowchart depicting the total number of articles obtained, the non-duplicate articles screened, the excluded articles (by title/abstract, full text screen , data extraction ) and finally the number of articles retrieved PRISMA: Preferred Reporting Items for Systematic Reviews and Meta-Analyses

A total of 106 articles were obtained from keyword search (based on PICO), and 91 manuscripts were retrieved after excluding the duplicates. Out of these 57 manuscripts were excluded on the basis of title and abstract. From the remaining 34 studies, 17 were excluded after reading the full text based on the inclusion and exclusion criteria [18-34]. During data extraction, four studies were removed and finally, 13 studies were included in this research (Figure 1).

The excluded studies and their reason for exclusion are described and tabulated (Table 1). 

**Table 1:** Articles excluded from this review and the reasons for exclusion

Author & Year	Reason for Exclusion
Scheller et al., 1998 [18]	Different outcome parameter
Louis et al., 2000 [19]	Different outcome parameter
Shinzato et al., 2000 [20]	Different outcome parameter
Hisatome et al., 2002 [21]	Different outcome parameter
Prissel et al., 2014 [22]	Different outcome parameter
Ganau et al., 2018 [23]	Different outcome parameter
Eid et al., 2021 [24]	Different outcome parameter
Moore et al., 2001 [25]	Different intervention group
Yukna et al., 2003 [26]	Different intervention group
Yang et al., 2012 [27]	Different intervention group
Bai et al., 2015 [28]	Different intervention group
Sandow et al., 2016 [29]	Different intervention group
De Vasconcellos et al., 2021 [30]	Different invention group
Calongne et al., 2001 [31]	Case Series
Girard et al., 2002 [32]	Case Report
Lithner et al., 2011 [33]	Review article
Asavamongkolkul et al., 2012 [34]	Different comparison group

The data of the selected studies were extracted using abstraction tables [35-47]. Information extracted from each study consisted of the Author and year, Study design, Duration, Intervention Groups and Sample size, Statistical methods, Outcome measures (Table 2).

**Table 2:** Table containing the data extracted with information on study design, intervention groups, sample size, statistics used and outcome measures of all included articles PMMA: Polymethyl methacrylate; UHMWPE: Ultra high molecular weight polyethylene; HA: Hydroxyapatite; MAT1: Material 1 (Ampreg 26; SPSystems Limited, Cowes, UK); MAT1-HA: Hydroxyapatite-coated material 1; MAT2: Material 2 (CG5052; Ciba Geigy Limited, Cambridge, UK); MAT2-HA: Hydroxyapatite-coated material 2; TiA1V-HA: Hydroxyapatite-coated titanium alloy; CT: Computed Tomography; PLGA: Poly (lactic-co-glycolic acid); PHEMA: Poly(2-hydroxyethylmethacrylate); PPF: Poly(propylene fumarate); α-TCP: α-Tricalcium Phosphate; HAP: Hydroxyapatite; DCP: Dicalcium Phosphate; PCL: Polycaprolactone; FA: Fluorapatite; GO: Graphene Oxide

Author & Year	Study Design	Time Period	Intervention Groups & Sample Size	Statistics	Outcome Measures
Goodman et al., 1994 [35]	Animal study	4 months	Preformed PMMA plu g; Doughy PMMA implant; Cement polymer powder; UHMWP plug; UHMWPE particles 15.68 μm (n=40)	Not Mentioned	Bone accretion
Zhang et al., 1999 [36]	Animal study	3 weeks	MAT1 (n= 18 ); MAT1-HA (n=24); MAT2 (n=24); MAT2-HA (n=36); TiA1V-HA (n=12); PMMA (n=12)	Student's t test	Mechanical testing Fluorescent label incorporation
Dean et al., 1999 [37]	Animal study	6 weeks	Textured PMMA ; Textured PMMA coated PLGA (n=23)	Student t test	Histomorphometric analysis new bone formation
Bruens et al., 2003 [38]	Retrospective study	20 years	Porous PMMA (n=24)	Not mentioned	Questionnaire Physical examination Transcranial CT
Giavaresi et al., 2004 [39]	Animal study	12 weeks	PMMA/HA/Glass; PMMA/HA/Glass + PHEMA (n=4)	Wilcoxon T-tests, Mann-Whitney U Monte Carlo methods	push-out force (Fmax) Affinity Index(AI)
Tsukeoka et al., 2006 [40]	Animal study	8 weeks	Zimmers bone cement; Modified PMMA bone cement ( n=4)	Mann Whitney U test	Push out test Micro CT
Kreigel et al., 2007 [41]	Retrospective study	44 months	PMMA Autograft (Tutoplast ^TM ^process, Tutogen Medical, Germany) (n=61)	Student’s t-test Fisher’s Exact Test.	Degree of Resorption Cosmetic outcomes Mean operating time
Hautamaki et al., 2008 [42]	Animal study	20 weeks	Porous glass fiber-reinforced prosthesis made of polymethylmethacrylate (PMMA) (n=19)	Student’s t-test Mann-Whitney U-test, ANOVA	Radiology, histomorphometry, scanning electron microscopy (SEM)
Kim et al., 2016 [43]	Animal study	8 weeks	Polyhydroxyethyl-polymethylmethacrylate (PHEMA-PMMA) membrane (n=18)	Mann-Whitney & Krunskal-Wallis test	Radiographic and histological analysis of bone regeneration.
Wu et al., 2017 [44]	Animal study	6 months	PMMA, PPF/a-TCP/HAP, and PPF/TtCP/DCP cements and without any filler control group (n=12)	ANOVA	Histologic and Radiographic examination (CT)
Pahlevanzadeh et al., 2018 [45]	In vitro study	28 days	PMMA-PCL; PMMA-PCL/FA; PMMA-PCL/GO; PMMA-PCL/FA/GO	Not mentioned	Bioactivity Compressive strength Elastic Modulus Yield strength
Cimatti et al., 2018 [46]	Animal study	3-6 months	PMMA-based porous cement (n=12); PMMA-based solid v/s porous cement (n=36)	ANOVA , Holm–Sidak post-test Kruskal–Wallis & Dunn post-test	Organ toxicity, coagulation tests, MRI images, radiography, micro-CT, SEM
Chung et al., 2021 [47]	In vitro study	16 weeks	poly(methyl methacrylate-co-3-(trimethoxysilyl)propyl methacrylate)-star- Silicon dioxide inorganic:organic (wt%); 50:50, 40:60, and 30:70 (n=6)	ANOVA Tukey's post hoc test	Cytotoxicity and Pre-Osteoblast Adherence Evaluations on Hybrid Scaffolds

The statistical significance, mean values of outcome measures and conclusion of each retrieved article were extracted (Table 3).

**Table 3:** Details of the author, year, outcome parameters of interest, mean values, statistical significance and author's conclusion of the studies included PMMA: Polymethyl methacrylate; BMR: Bone mineralization rate; PLGA: Poly(lactic-co-glycolic acid); HA: Hydroxyapatite; PHEMA: Poly(2-hydroxyethylmethacrylate); SBF: Simulated Body Fluid; AI: Affinity Index.

Author & Year	Outcome Parameter of Interest	Mean±SD	P-value	Author’s Conclusion
Goodman et al., 1994 [35]	Bone accretion	Not mentioned	Not mentioned	Doughy PMMA suppressed bone formation, preformed PMMA plugs and particulate PMMA polymer did not.
Zhang et al., 1999 [36]	Fluorescent label incorporation	PMMA (3.12 ± 0.59 mm/day)	p<0.01	Bone mineralization rate (BMR) after 3 weeks of implantation: no significant differences between PMMA and uncoated materials
Dean et al., 1999 [37]	Histomorphometric analysis new bone formation	1.11 x 10 6 0.78 um2 -1.77x 10 6 0.74 um2	p<0.038	At 6 weeks, PMMA discs coated with PLGA and periosteum showed good new bone formation
Bruens et al., 2003 [38]	Transcranial CT	Not mentioned	Not mentioned	There were no side effects to the porous PMMA. CT scans showed bone ingrowth in the prostheses.
Giavaresi et al., 2004 [39]	push-out force (Fmax) Affinity Index(AI)	12 weeks (Fmax) PMMA/HA/Glass- 96 ± 12 PMMA/HA/Glass+pHEMA - 23± 3 AI between the experimental times (4 versus 12 weeks) was observed only in the PMMA/HA/Glass+pHEMA implants (22%) PMMA/HA/Glass+pHEMA versus PMMA/HA/Glass: 4 weeks= 33%, 12 weeks= 19%	p < 0.001	Good mechanical and histomorphometric results with PMMA/HA/Glass positive effect of SBF on pHEMA and to enhance the coating adhesion.
Tsukeoka et al., 2006 [40]	Push out test Micro CT	1.60MPa in 3 weeks for modified PMMA	p=0.02	Higher binding strength and percentage fraction of the calcified bone in the modified PMMA bone cement than in Zimmers cement.
Kreigel et al., 2007 [41]	Degree of Resorption	Tutoplast® cases. Global resorption occurred in 11.8%	p = 0.005	In adults, Tutoplast® processed bone autografts is an alternative to the standard PMMA, especially for large craniotomy defects.
Hautamaki et al., 2008 [42]	One-way analysis of variance (ANOVA), Mann-Whitney U-test, and Student’s t-test	Bone contact index (BCI) at the posterior cortex was higher for PMMA at 20 weeks than control group	p=0.01	Porous surface enhanced ingrowth of host bone
Kim et al., 2016 [43]	Radiographic and histological analysis of bone regeneration.	Control -34.8±12.8 Experimental -50.3±13.7	p<0.05	PHEMA-PMMA is a potential material for guided tissue regeneration membrane. No adverse tissue reaction and good bone regeneration.
Wu et al., 2017 [44]	Histologic sections CT images	In 10/12 samples, direct incorporations of bone with the PMMA block were observed and only 2 had fibrous tissue interposition	Not mentioned	CPC/PPF - a promising option to replace PMMA
Pahlevanzadeh et al., 2018 [45]	Bioactivity	Ca/P molar ratio: PMMA-PCL/FA -1.60 PMMA-PCL/FA/GO -1.62	p<0.05	PMMA-PCL/FA/GO bone cement: Favorable bioactivity, Suitable mechanical properties, and high cell viability
Cimatti et al., 2018 [46]	Radiography, micro-CT, SEM	20.81 ± 5.88% bone formation	p<0.05	6 mm linear ingrowth from the bone–cement interface 20% bone ingrowth in the whole defect area.
Chung et al., 2021 [47]	Cytotoxicity and Pre-Osteoblast Adherence	% of new bone volume relative to total defect volume (BV/TV): S60 group -12.4%, control group of 19.6%	p<0.01	Hybrid scaffolds with 40:60 inorganic:organic composition induce new vascularized bone formation

Discussion

The current review included 13 articles after exclusions and the core data was tabulated and assessed. The analysis describes the use of resin bone graft substitutes in various defects. Out of the 13 studies, only two studies involved human subjects which were retrospective in nature (Table 2). The majority of the included articles are in vitro studies or studies on animal models, which highlights the novelty of the intervention group studied [35-47].

PMMA as a bone graft has been sporadically used as graft material due to its desirable injection properties and sufficient mechanical properties. Research in this aspect has established favorable bioactivity, high cell viability, potential guided tissue regeneration membrane, and bone ingrowth into the prostheses [38,42].

However, certain shortcomings such as questionable efficiency in the biological environment, increased temperature, and toxicity due to monomers do exist. Various studies tried to alter PMMA bone grafts by surface coating them with polydopamine-strontium calcium polyphosphate (D/SCPP), titanium dioxide, calcium phosphate, antibiotics, etc. to overcome its clinical disadvantages.

Another important problem regarding the PMMA-based graft is the interface between bone and cement which tends to be a weak zone. This essentially is the reason for the development of PMMA coatings, doped PMMA, and composite graft solutions [39,40]. Time and again in vitro models and animal studies have shown promising evidence that PMMA can be used in defect coverage, especially craniofacial defects with a set of challenges [48,49].

The limitations of this study comprise the in-vitro and animal model-based studies included with level V evidence (according to the Centre for Evidence-Based Medicine, Oxford, England) and the lack of randomized control trials in humans with the intervention or comparison groups. Therefore, the inference must be perceived cautiously. The future scope of this research could include multicentre trials in humans with more randomization and stricter protocols for consideration.

## Conclusions

Based on the analysis of this review, we could conclude that polymethylmethacrylate is a very potential bone substitute for 3-dimensional reconstruction of maxillofacial defects. However long-term clinical trials are required for rigorous validation. With the alloplastic bone graft materials being an advanced grafting solution option for various defects, they may be a likely substitute in the near future.
